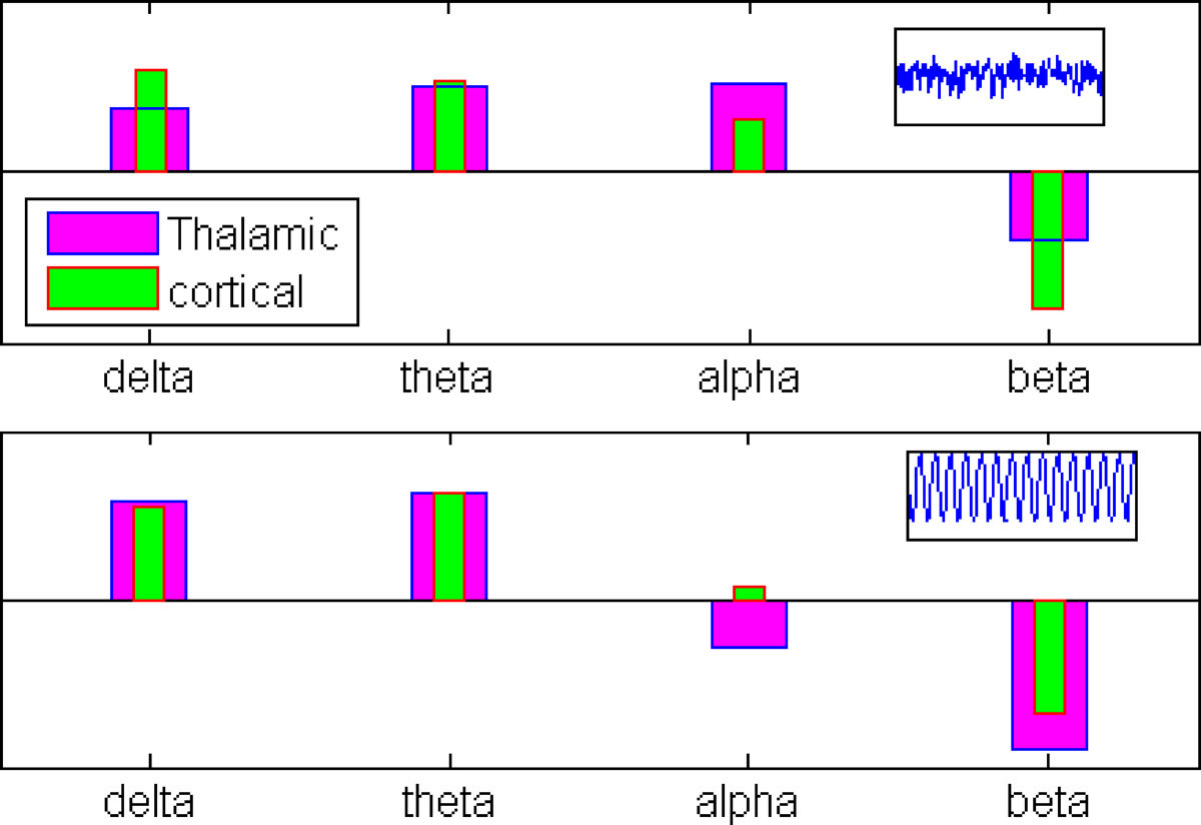# Combined study of time-series bifurcation and power spectral behaviour of a thalamo-cortico-thalamic neural mass model

**DOI:** 10.1186/1471-2202-14-S1-P18

**Published:** 2013-07-08

**Authors:** Basabdatta Sen-Bhattacharya

**Affiliations:** 1 School of Engineering, University of Lincoln, Lincoln, LN6 7TS, UK

## 

A combined power spectral and time-series bifurcation analysis of a neural mass model is presented. Such 'multi-modal' analytical techniques are being used in several researches to understand Electroencephalograph (EEG) anomalies in brain disorders [1][2], in contrast to 'power spectra-only' analytical studies that were more common during the early days of EEG analysis. In a recent work, a combined analysis of a simple thalamo-cortical neural mass model in context to EEG abnormality in Alzheimer's disease (AD) is presented [3]. The study shows that 'unimodal' analytical techniques such as power spectra-only studies without a simultaneous observation of the time-series model output may lead to anomalous conclusions and hypotheses. Towards this, in this work, a 'multi-modal' analytical technique is applied on a thalamocorticothalamic (tct) model, which was earlier studied using power-spectra analysis only [4]. The tct model is an enhanced version of that used in [3] and is based on biological data available in current literature. Furthermore, it aims to mimic thalalmocortical oscillations such as observed in the EEG of both healthy and diseased brain.

Here, the power spectra of the tct model output is observed within the δ (1-3 Hz), θ (4-7 Hz), α (8-13 Hz), β (14-30 Hz) bands, along with a simultaneous analysis of the time series behaviour, the latter showing three behavioural modes: noisy point-attractor, spindle and limit-cycle. With all parameters at their basal values, the output time series is in a noisy point-attractor mode with maximum power within the alpha band (Figure 1). However the model shifts into a limit cycle oscillatory mode with a decrease in inhibitory connectivity parameters in the model (Figure 1); the corresponding power spectra show an increase in peak power within the θ and δ bands along with a simultaneous decrease in power within the α and β bands. The model behaviour is very much in agreement with in-vitro studies [5] which report an increased theta band power and a simultaneous decreased alpha band power during transition from wakefulness to sleep. Furthermore, the in-vitro time-series are qualitatively very similar to those obtained using the model. Thus, the model indicates a decreased inhibitory activity to be the neural correlate of the transitive state between wakefulness and sleep. On the other hand, increased mean firing activity of the extrinsic model inputs pushes the model, first into a spindling mode, and then into a limit cycle mode. In this state, the power within the delta band shows a significant increase compared to those within the other frequency bands. This behaviour is more similar to in-vivo studies of awake-to-sleep transition as reported in [5].

**Figure 1:**